# Impact of socioeconomic position and distance on mental health care utilization: a nationwide Danish follow-up study

**DOI:** 10.1007/s00127-017-1437-2

**Published:** 2017-08-28

**Authors:** Aake Packness, Frans Boch Waldorff, René dePont Christensen, Lene Halling Hastrup, Erik Simonsen, Mogens Vestergaard, Anders Halling

**Affiliations:** 1 Research Unit of General Practice, Institute of Public Health, University of Southern Denmark, J.B. Winsløws Vej 9A, 5000 Odense C, Denmark; 2 Psychiatric Research Unit, Region of Zealand, Slagelse, Denmark; 3 Department of Public Health, Institute of General Medical Practice, Aarhus University, Aarhus, Denmark; 4 Centre for Primary Health Care Research, Lund University, Lund, Sweden; 5 Faculty of Health and Medical Sciences, Institute of Clinical Medicine, University of Copenhagen, Copenhagen, Denmark

**Keywords:** Socioeconomic factors, Mental health services, Access to health care, Antidepressants, Geographic information system

## Abstract

**Purpose:**

To determine the impact of socioeconomic position (SEP) and distance to provider on outpatient mental health care utilization among incident users of antidepressants.

**Method:**

A nationwide register-based cohort study of 50,374 person-years.

**Results:**

Persons in low SEP were more likely to have outpatient psychiatrist contacts [odds ratio (OR) 1.25; confidence interval (CI) 1.17–1.34], but less likely to consult a co-payed psychologist (OR 0.49; CI 0.46–0.53) and to get mental health service from a GP (MHS-GP) (OR 0.81; CI 0.77–0.86) compared to persons in high SEP after adjusting for socio-demographics, comorbidity and car ownership. Furthermore, persons in low SEP who had contact to any of these therapists tended to have lower rates of visits compared to those in high SEP. When distance to services increased by 5 km, the rate of visits to outpatient psychiatrist tended to decrease by 5% in the lowest income group (IRR 0.95; CI 0.94–0.95) and 1% in the highest (IRR 0.99; CI 0.99–1.00). Likewise, contact to psychologists decreased by 11% in the lowest income group (IRR 0.89; CI 0.85–0.94), whereas rate of visits did not interact.

**Conclusion:**

Patients in low SEP have relatively lower utilization of mental health services even when services are free at delivery; co-payment and distance to provider aggravate the disparities in utilization between patients in high SEP and patients in low SEP.

**Electronic supplementary material:**

The online version of this article (doi:10.1007/s00127-017-1437-2) contains supplementary material, which is available to authorized users.

## Introduction

In a health care system responding adequately to need, patients in most need would be expected to receive more health care service and more specialized care. Inequalities in health and the ability of health care systems to address this issue remain of concern in European countries [1].

A study of OECD countries concludes that people with higher incomes are significantly more likely to see a specialist than people in lower SEP [2]. This is supported by population surveys in Denmark which show a linear correlation between increasing education and increasing use of specialist services [3]. In Holland, the same pattern exists as the more educated people are less likely to use primary care in the event of emotional problems and more likely to use mental health care services compared to people with shorter education [4]. Since common mental health problems are significantly more frequent in populations in lower SEP [5, 6], the utilization of services would be expected to reflect this. However, surprisingly it does not. It could be argued that distance to the services may explain the difference in use, since the specialists primarily live and practice close to people in high SEP [7]. Indeed, distance to mental health services matters.

The impact of distance on the utilization of mental health care services has been subject to analyses for more than 150 years. In 1853, Edgar Jarvis described how the utilization of mental hospitals was inversely proportional to the travel distance in the catchment area [8]. This has been proven repeatedly since then and has also been shown to be relevant for outpatient treatments [9] and within cities too [10]. Compared to somatic health care, the utilization of mental health care services is more sensitive to travel distance [11]. Distance has an impact on the type of treatment chosen by patients with depression, as longer distance is associated with less therapy and more antidepressants and thus sub-standard treatment [12, 13]. In Australia, distance to mental health services has proven to be a barrier in itself, affecting persons in low SEP more strongly [14].

Knowing that SEP and distance to mental health services are of importance to utilization makes it likely that the remote areas would be underserved. The Inverse Care Law, stating that remote areas are drained for jobs, healthy citizens, and subsequently health services, is an issue of concern [15]. In fact, ecological data show that the remote and most deprived municipality in Denmark received 20% less outpatient mental health care services in 2013 than what would be expected for the population size (psychologist, private or public psychiatry; unpublished data). Except for the Australian study mentioned, no previous studies had examined the socioeconomic impact of distance to outpatient mental health service utilization at an individual level.

The aim of the study is to determine the impact of socioeconomic position and distance to provider on outpatient mental healthcare utilization among incident users of antidepressants.

## Method

### Study design

The study was conducted as a register-based one-year follow-up study on mental health service utilization after initiated treatment with antidepressants.

### Settings

The Danish health care system is tax-funded and free at delivery for both primary and secondary care except for dental care and treatments at psychologists, which are only partly subsidized [16]. The general practitioner (GP) has a gatekeeper function, and specialized care is only free after referral. Treatment by a psychologist is subsidized for patients referred from a GP, for some specific conditions: reaction to specific traumatic events, mild to moderate depression and, specifically, for citizens between 18 and 38 years old, also mild to moderate anxiety disorders. In 2014, the down payment was equivalent to 52€ for the first consultation and 44€ for the following sessions [17]. The psychologist needs a special authorization by The Danish Supervisory Board of Psychological Practice in order to be subsidized.

### Study population and study period

The study population consisted of all individuals aged 20–64 years living in Denmark who were prescribed antidepressants (Anatomical Therapeutic Chemical (ATC) classification system N06A) in 2013, according to data extracted from The Danish National Prescription Registry [18, 19]. Only patients with no previous prescription of antidepressants in 2012 were included. Bupropion (ATC N06AX12) was not included since it is only prescribed for smoking cessation in Denmark. Tricyclic antidepressants (ATCs N06AA) were not included either as they are not recommended as the first choice for treatment of depression and are frequently used as a secondary analgesic [20, 21]. All persons migrating in 2012 were excluded as they could not be accounted for during the full study period. Finally, all patients coded as terminally ill at first prescription, and thereby specially subsidized, were excluded [22]. The resulting population was followed for 12 months per individual.

All persons with permanent residence in Denmark are registered in the Danish Civil Registration System (CRS) [23]. They are assigned a unique 10-digit personal identification number, called the CPR number (Central Personal Register Number). By this number, it is possible to identify an individual in all public registers.

### Independent variables

Data on family income were drawn from the Danish registers on personal income and transfer payments [24] from Statistics Denmark [25]. Family income was chosen since the household represents shared common resources, and because, as far as income is concerned, it is more strongly and consistently associated with health than individual income [26]. In this study, we used equivalent disposable family income. (see Supplement).

Highest completed educational level was drawn from the Population’s Education Register [27].

The home addresses of the study population were drawn from CRS and GIS positioned (geographic information system). Addresses for all GPs, psychologists and private psychiatrists were drawn from The Danish National Health Provider Register. Addresses for outpatient mental health care services (public psychiatric services) were drawn from homepages and confirmed by regional officials. The distances in metres by road from the participant’s home address to the nearest located health provider at the time of the first prescription have been calculated by Statistics Denmark in ESRIs ArcMap 10.3 using Network Analyst.

Access to a motorized vehicle was verified through The Digital Motor Register, Statistics Denmark. If a vehicle was registered to an individual in the study population or a member of the family, it was considered as positive access. Vehicle registration was categorized into none, car owners, motorcycle and 45 mopeds. If a car and a motorcycle and/or 45 mopeds were owned by the same person or family, only the car was included.

Data concerning age, sex, address, marital status, cohabitation status, country of origin and vital status were gathered from the CRS.

Country of origin was grouped into (1) Denmark; (2) the EU and other European countries, North America and Oceania as Europe/Western countries; and (3) Africa, South and Latin America, stateless and unknown as non-western countries.

Information on comorbidity was drawn from The Danish National Patient Register [28] and The Danish Psychiatric Central Research Register [29] (see Supplement). These registers provide information on morbidity and comorbidity in secondary health care.

Information on psychiatric comorbidity was obtained for patients who had received inpatient or outpatient hospital services.

### Dependent variables

Data on the utilization of private psychiatrist, psychologist and general practitioner (GP) were drawn from The Danish National Health Service Register for Primary Care [30] (see Supplement).

Only mental health services by GPs (GP-MHS) were analysed. GP-MHS covers talk therapy by a GP. It consists of at least two talks within the first 6 months and not more than seven talks within 1 year. The service triggers additional pay.

Information on public inpatient and outpatient psychiatric treatment was drawn from The Danish National Patient Register; ICD-10 coded F00–F99.

Data on outpatient public psychiatric services and services by private outpatient psychiatrists were grouped together in the analyses as public outpatient psychiatric services are used instead of private services, in areas with no access to a private psychiatrist. The grouping was termed outpatient psychiatrist.

One-day psychiatric hospital admissions were re-categorized into emergency contacts and termed emergency and short admissions.

The collection and handling of the data have been approved by The Danish Data Protection Agency J. no. 2015-41-3984. Approval by an ethic committee is not required for register studies.

## Statistical analyses

Logistic regression was used to calculate the odds ratio (OR) for the association between SEP and contact to a health service provider. Among those who had contact to a mental health service provider, Poisson regression was used to calculate the incidence rate ratio (IRR) for the association between SEP and the frequency of contacts. Both analyses were adjusted for gender, age, cohabitation status, country of origin, somatic as well as psychiatric comorbidity, and access to a vehicle.

A logistic as well as a Poisson regression analysis of interaction between income and distance, and education and distance, was performed for each outcome measure. For interactions significant at a level of 0.01 or less, further analyses were performed; the impact of distance on contact to the identified mental health service was analysed by logistic regression on income and/or education stratified within groups. Distance was measured in 5 km intervals. The analysis of the impact of distance within different educational and/or income groups on the frequencies of contacts was done by Poisson regression. These analyses were done for each type of health care service showing interaction.

OR and IRR were estimated at 95% confidence intervals (CI), and p-values were reported.

## Results

We followed a cohort of 50,636 incident users of antidepressants for 50,374 person-years at risk. Nearly 60% of the study population were female, and 50% were older than 41 years. The age distribution was close to that of the national distribution (Table 1).

**Table 1:** Characteristics of the study population

	Total	
	*N*	Pct
Gender	50,374	
Male	21,736	43
Female	28,638	57
Age at entrance		
20–29	11,065	22
30–39	11,750	23
40–49	12,734	25
50–59	10,819	21
60–64	4006	8
Family type		
Single	21,769	43
Cohabitating	28,605	57
Education		
<10 years	16,256	32
10–12 years	21,100	42
>12 years	10,827	21
NA	2191	4
Country of origin		
Denmark	42,519	84
Europe and Western countries	4137	8
Non-western countries and unknown	3718	7
Vehicle		
None	29,387	58
Car	20,375	40
MC	320	1
45 moped	292	1
Comorbidity, somatic		
Cancer (latest 10 years)	1467	3
Diabetes	1333	3
Ischaemic heart disease	2881	6
COPD	720	1
Arthrosis	484	1
No chronic somatic		
0	44,308	88
1	5308	11
2	698	1
3	59	0
4	1	0
Comorbidity psychiatric		
Former mental disorder	12,027	24

*MC* motor cycle, *COPD* chronic obstructive pulmonary disease, *Chron* chronical diseases

A total of 9476 individuals (19%) of the study population used services provided by psychologists within the one-year follow-up (Table 2). Among persons in contact with public psychiatrists, 603 (9%) were in contact with private psychiatrists, and 1143 persons (16%) were in contact with a psychologists (not shown).

**Table 2:** Total number of contacts to mental health care services and distance to outpatient services

Type of health care service used	*N*	Pct	Total sum of contacts
Public psychiatrist (outpatient mental health clinic)	7035	14	75,209
Admission mental hospital >1 day	1783	4	2619
Psych. emergency ward = <1 day	1811	4	2599
Private psychiatrist	4681	9	31,279
Psychologist	9476	19	64,865
GP-MHS	17,638	35	56,692
GP consultation	48,711	97	3,72,265
Person-years			50,374

Distance to outpatient provider in kilometres					
Type	Mean	Median	90%	Min	Max
GP	2.1	1.1	5.6	0	26.3
Psychologist	4.4	2.1	12.0	0	56.0
Private psychiatrist	10.6	4.7	25.6	0	191.9
Public psychiatrist	10.7	6.7	25.6	0	87.2
Outpatient psychiatrist^a^	7.8	3.8	19.9	0	85.6

*GP* general practitioner, *GP-MHS* GP mental health services, equivalent to talk-therapies provided by GP

^a^Outpatient psychiatrist combines public psychiatrist and private psychiatrist—distance calculated to the nearest one

### SEP and contact and rates of contact to mental health care services

Persons with the lowest incomes established contact to outpatient psychiatrists more often (OR 1.25; CI 1.17–1.34) compared to persons in the highest income group (Table 3); contact to a psychologist was less for lower income groups (OR 0.49; CI 0.46–0.53) and fewer years of education (OR 0.37; CI 0.35–0.40), compared to higher income and educational groups. The same picture was seen for contact to GP-MHS as for psychologist related to income (OR 0.81; CI 0.77–0.86) and to education (OR 0.71; CI 0.67–0.75) compared to the highest groups.

**Table 3:** Incidence rate ratios of contact among patients who had one visit or more

Poisson	Outpatient psychiatrist					Psychologist					GP-MHS					Psych. emergency clinic					Admissions				
Family equivalent income^a^	IRR	*p*	CI		*N*	IRR	*p*	CI		*N*	IRR	*p*	CI		*N*	IRR	*p*	CI		*N*	IRR	*p*	CI		***N***
Highest third	1				11,113	1				9033	1				17,638	1				1752	1				1783
Middle third	**0.90**	**<0.001**	**0.88**	**0.91**		**0.93**	**<0.001**	**0.91**	**0.95**		1.00	0.639	0.98	1.02		1.02	0.734	0.91	1.15		1.03	0.614	0.92	1.14	
Lower third	**0.83**	**<0.001**	**0.81**	**0.84**		**0.94**	**<0.001**	**0.91**	**0.96**		**0.94**	**<0.001**	**0.92**	**0.97**		1.06	0.364	0.94	1.14		0.95	0.382	0.84	1.07	
Education^a^																									
12+ years	1				10,659	1				8869	1				17,038	1				1677	1				1709
years 10–12	**0.92**	**<0.001**	**0.90**	**0.93**		**0.92**	**<0.001**	**0.90**	**0.93**		0.99	0.395	0.97	1.01		0.98	0.729	0.87	1.10		1.00	0.988	0.89	1.12	
<10 years	**0.75**	**<0.001**	**0.74**	**0.76**		**0.80**	**<0.001**	**0.79**	**0.82**		**0.93**	**<0.001**	**0.91**	**0.96**		1.06	0.370	0.94	1.19		1.04	0.511	0.92	1.17	

Two separate analyses of correlation of income or education with type of mental health service used

Psychiatric emergency clinic includes emergency contacts and admissions up to 1 day

Results significant within a 95% confidence interval are marked in bold

^a^Adjusted for age, gender, country of origin, cohabitating status, access to vehicle, comorbidity psychiatric, comorbidity somatic

*GP-MHS* mental health services by general practitioner (talk therapy), *Admission MH* admission to mental hospital, *IRR* incidence rate ratio, *CI* confidence interval, *p* 0.05

No significant association with education or income and contact to emergency or inpatient psychiatric services was found.

Among patients who had contact to mental health care services, persons in lower SEP had lower rates of visits to outpatient psychiatrist (Income IRR 0.83, CI 0.81–0.84; education IRR 0.75, CI 0.74–0.76), psychologist (Income IRR 0.94, CI 0.91–0.96; education IRR 0.80, CI 0.79–0.82) and visits to GP-MHS (Income IRR 0.94, CI 0.92–0.97; education IRR 0.93, CI 0.91–0.96) compared to those in higher SEP when adjusted for socio-demographics, comorbidity and access to a vehicle (Table 3).

Rates of contact to emergency or inpatient psychiatric services did not differ across SEP.

### Distance to outpatient mental health services

Distances to health care services were short for most persons (Table 2). The average distance was 2 km to a GP, 4.4 km to the nearest psychologist and 9 km to the nearest outpatient psychiatrist. Only 10% had more than 12 km to the nearest psychologist or more than 20 km to the nearest outpatient psychiatrist.

We found an interaction between income, education, distance and rate of visits to outpatient psychiatrists. The incidence rate ratio of contacts decreased by 1% for the highest and 5% for the lowest income group for each additional 5 km travel distance to an outpatient psychiatrist; likewise the rate decreased by 3% for patients with less than 10 years of education and 5% for patients with 10–12 years of education. There was no significant association between distance and use of outpatient psychiatrist among patient with the longest education (Table 4). There was no interaction between income, education, distance and contact versus no contact to outpatient psychiatrist.

**Table 4:** Impact of distance and income and education on mental health care utilization—stratified by SE groups

Outpatient psychiatrist				Psychologist			
Incidence rate ratio of contact^a^				Contact to health service y/n^a^			
Income	Each additional 5 km			Income	Each additional 5 km		
	IRR	CI	*p*		OR	CI	*p*
Highest income	0.99	(0.98; 1.00)	0.005	Highest income	0.98	(0.94; 1.02)	0.256
Medium income	0.95	(0.94; 0.95)	<0.001	Medium income	0.98	(0.94; 1.02)	0.299
Low income	0.95	(0.94; 0.95)	<0.001	Low income	0.89	(0.85; 0.94)	<0.001
Education	IRR	CI	*p*	Stratified log reg			
12+ years	0.99	(0.98; 1.00)	0.81				
10–12 years	0.95	(0.94; 0.95)	<0.001				
<10 years	0.97	(0.96; 0.98)	<0.001				

Stratified Poisson

*SE* socioeconomic, *OR* odds ratio, *IRR* incidence rate ratio, *CI* confidence interval; *p* 0.05

^a^Adjusted for age, gender, cohabitating status, country of origin, psychiatric emergency visits, comorbidity somatic, comorbidity psychiatric

We found interaction between income, distance and contact versus no contact to psychologist; contact decreased by 11% per additional 5 km travel distance for the lowest income group. The lowest income group was the only group significantly affected by distance, when adjusted for age, gender, cohabitating status, country of origin, psychiatric emergency visits, somatic and psychiatric comorbidity. We did not find interactions between income, education, distance and rates of visits to a psychologist, nor did we find interactions on contact or rates of visits to GP-MHS.

## Discussion

Overall, our large population-based cohort study showed that persons with short education or low income had significantly fewer mental health care visits during the year following a first prescription of antidepressants, compared to person with long education or high income. Persons with shorter education had fewer contacts to outpatient psychiatrists, psychologists and GP-MHS. Persons in the lowest income group were more likely to have contact to outpatient psychiatrists, but then their rates of visits were lower. Low income was associated with less contact to a psychologist and, to some extent, also with less mental health care services provided by the GP compared to high income.

Distances to all outpatient mental health services were short. It is notable that, concerning contact to service providers, only income and contact to psychologist showed interaction with distance. Distance was a socioeconomic differentiating obstacle to rates of visits to outpatient psychiatrists, but not to contact.

### Who are affected by this study?

The study population consisted of one-fifth of the 246,755 annual users of these antidepressants in the age group of 20–64 years in Denmark in the year 2013 [31]. By this selection, we expected to embrace patients with what is called common mental disorders (CMD) defined by the National Institute for Health and Care Excellence as depression and anxiety disorders, including OCD and PTSD, which may affect up to 15% of the population at any given time [32]. For all of these disorders, the recommended pharmacological treatment is antidepressants, if any [33]. These patients are often seen in general practice.

### Treatment by outpatient psychiatrists

Outpatient psychiatrists more often had contact to patients in the lowest income group than to patients in the highest income group, but the incidence rate ratios of contacts decreased in the lower income groups. Even though longer education was not associated with increased contact, the rates of visits to outpatient psychiatrist decreased in the shorter educational groups.

It is not likely that a higher need for outpatient psychiatric services should come with higher SEP, nor is it likely that the few patients in high SEP referred to mental health services are in more need when referred. We expected that prescriptions of antidepressants were based on symptoms and independent of SEP. While distance was found to have impact on rates of contacts to outpatient psychiatrists, these findings could also indicate a different therapeutic approach to persons in higher SEP. It is possible that persons in higher SEP had a shorter delay in referral to a psychiatrist, and thereby gained a wider timeframe for visits within the 12 months after initiated antidepressant treatment. Thus, this finding could be a possible result of the referral pattern by the GP.

### Treatments by a psychologist

Contact to a psychologist was strongly associated with SEP. We found a significant increase in utilization for each upward step in the income category and likewise for increasing length of education. The impact of income was most likely due to the required co-payment. Contacts to psychologists dropped significantly for the age groups above 40 in the study population (not shown), which underlines the economic impact as treatment of anxiety disorders is not subsidized beyond the age of 38. Additionally, it has been documented that co-payment is associated with disfavouring the lower income groups in the Danish health care system, as in other health care systems [34, 35]. More specifically regarding mental health services, it is stated that co-payments restrict access to outpatient mental health services regardless of need [36]. Part of the differences in utilization could also be due to easier access for patients with a private insurance, typically provided by an employer.

It has been argued that mental health therapies make heavy demands on the clients’ cognitive capacities, and this could increase the obstacles for people with less education [4]. This may explain some of the difference in utilization between highest and lowest educational groups, but probably not the difference from high to the middle income or middle educational group.

### The GP’s role

The GP is very accessible in Denmark, and mostly there are no waiting periods. There are clinics close by, and the service is free at delivery. The GP could potentially compensate for social inequality in the use of mental health care by giving more therapeutic consultations to patients in low SEP. However, we did not find evidence of this as the GP offered less mental health services to people in low SEP. In addition, the frequency of MHS (talk therapy) offered by the GP was lower among patients in low SEP.

The GPs were in contact with 97% of the study population during the year following the initial prescription, and 35% received GP-MHS (Table 2). Relevant methods are expected to be used, when a GP performs MHS, but it has not been documented which ones are actually used [37]. In this study, 45% had two GP-MHS visits or less (not shown), which could indicate that a supportive approach was common.

### Comparison with other studies

We have compared our findings with population studies from European countries, where some kind of estimation of need has been associated with SEP and the utilization of mental health services.

In a Norwegian questionnaire-based, cross-sectional population study, income was not associated with outpatient visits to a psychiatric clinic, among those who reported anxiety/depression. Higher education, however, was associated with more frequent contact (OR for trend 1.34; 1.08–1.68) [38]. Being nationwide and fully comprehensive of service utilization, we consider our study reliable.

A population study from the Netherlands focused on CMD severity and treatment contact to mental health care (MHC) and general medical care. They found that 12 months of treatment with contact to MHC was less frequent for shorter educated persons, and that income had no impact on contact. The rates of visits to MHC were related to the severity of the mental disorder, while the rates of visits to general medical care were not. There were no sociodemographic characteristics related to the highest treatment frequency, not even after adjusting for the disorder severity. 40% of the MHC users did not have a 12-month disorder, and 39% of the persons with severe disorders did not have contact to MHC [39]. In the Netherlands, access to MHC is free of charge, which could explain the difference to our findings, if both psychiatrist and psychologist had been pooled together.

A study from the UK, describing the impact of SEP on psychotherapy use, had similar findings to ours. They studied patients with treatment needs defined as common mental disorder based on a 12-item General Health Questionnaire (GHQ-12). The use of private psychotherapists was closely associated with higher education (OR 3.08–6.51) and highest income groups (OR 1.65–3.33), as compared to the lowest. Co-payment ranged from 40 to 100£ per session. The use of public psychotherapists was lower for the highest income groups and the highest educational group. In the study, psychotherapists also included psychiatrists and (psycho-)analysts [40]. The finding of high SEP being associated with the use of private psychotherapy was similar to our study, given that the term psychotherapist is equivalent to psychologist. Our anticipated socioeconomic impact of co-payment finds support in this study.

To our knowledge, there are no other studies of the combined impact of SEP and distance on the utilization of mental health services, so a comparison with other studies was not possible.

Among the strengths of this study were the nationwide selection of patients with a professionally evaluated need for antidepressants drugs and the possibility of following their subsequent treatment for 1 year without loss to follow-up. By this method, it was possible to detect not only the users of mental health services but also the non-users, among incident users of antidepressants.

The comprehensiveness of the national registers on social and health data was a strength. The validity and completeness of the outcome data from The Danish National Health Service Register for Primary Care is high [30]. Because the data are connected to reimbursement, the coverage is assumed to be good. Data gathered from continuously updated registers are independent of memory errors and free of recall bias.

We were able to identify actual GIS-positioned distances by road to the nearest outpatient psychiatrist, psychologist and GP at an individual level for all but 301 persons (0.6%) and thereby gained precise and reliable data on distance to the services. We combined this with SEP, which, to our knowledge, has not been done before.

There were some limitations of this study. Our selection of study population is based on patients receiving antidepressants. If the prescription pattern differs, and individuals in high SEP more often use psychologist services instead of antidepressants, they would not be included in the selection. This could partly explain the high proportion of persons with a short education in our study. If this potential selection bias was present, it would aggravate the unequal use of mental health services found, whereas it would not have an impact on the evaluation of the effect of distance.

Distance is relative to time travelling. A short distance in a large city may require longer time to cover than the same distance elsewhere. At some places using public transport is faster than using a car and vice versa. The study could have obtained higher precision on the obstacle of travel, if travel time by car and public transport were obtained and combined. Unfortunately, travel time by public transport was not accessible at Statistics Denmark.

The distance was measured to the nearest outpatient psychiatrist/psychologist/GP, but not to the ones actually used. Except for waiting periods for the GP, waiting periods could be an obstacle for access. The general waiting periods for private and public psychiatrists were 4–6 weeks in 2013 [41], whereas the general waiting period for psychologist were 9–10 weeks [42]. The “true” impact of distance could be blurred by the effect of waiting periods, especially if the services are associated with additional barriers as e.g. co-payment. The more affluent patients would probably not wait and would be willing and capable to pay for a specialized service by a psychologist or to travel to services further away. Thus, the socioeconomic difference in contacts to mental health care services seen in the study could be explained by the additional distance to accessible services affecting people in low SEP stronger. The fact that we did not find distance of importance to contact to outpatient psychiatrist, but only to rates of visits, shows a limit to this residual confounder.

The full impact of distance on mental health services utilization is probably not revealed in this study. Distance could still be a serious local problem. Spatial analyses would be a more potent method to analyse the impact of distance since all localities would be shown by this method, and the density of services could be accounted for as well [43].

In summary, we found that higher SEP was strongly associated with contact to outpatient mental health services and with higher rates of contacts, overall. Psychiatric services were used more by the less affluent patients, but used more frequently by patients in high SEP. The psychologist services were used more by patients in high SEP, as were GP-MHS.

Increasing distance to a health care provider did show a modest adverse socioeconomic impact on service utilization, in a national setting with short distances to mental health services.

### Clinical recommendations

The social inequality in the utilization of mental health services seen in this study calls for actions. The GP-MHS could be directed towards patients in lower SEP to a higher extent.

The initial psychiatric evaluation may be at a distance from patients home, but treatment requiring frequent attendance ought to be closer to the residence of the patients in low SEP.

### Policy recommendation

The grave socioeconomic imbalance in the utilization of psychologist services does not correspond to a health service aiming at equal treatment to equal need. Access to psychologists free of charge would improve social equality in health care treatment considerably.

## Electronic supplementary material

Below is the link to the electronic supplementary material.

Supplementary material 1 (DOCX 24 kb)
