# Correction to: Impact of socioeconomic position and distance on mental health care utilization: a nationwide Danish follow-up study

**DOI:** 10.1007/s00127-018-1556-4

**Published:** 2018-07-10

**Authors:** Aake Packness, Frans Boch Waldorff, René dePont Christensen, Lene Halling Hastrup, Erik Simonsen, Mogens Vestergaard, Anders Halling

**Affiliations:** 1 Research Unit of General Practice, Institute of Public Health, University of Southern Denmark, J.B. Winsløws Vej 9A, 5000 Odense C, Denmark; 2 Psychiatric Research Unit, Region of Zealand, Slagelse, Denmark; 3 Department of Public Health, Institute of General Medical Practice, Aarhus University, Aarhus, Denmark; 4 Centre for Primary Health Care Research, Lund University, Lund, Sweden; 5 Faculty of Health and Medical Sciences, Institute of Clinical Medicine, University of Copenhagen, Copenhagen, Denmark

## Correction to: Soc Psychiatry Psychiatr Epidemiol (2017) 52:1405–1413 10.1007/s00127-017-1437-2

In the original publication of this article, Table 3 was published incorrectly. The corrected table is shown below.

**Table 3:** Separate analyses of correlation of income or education with mentalt health service used, in odds ratios and incidence rate ratios of contact

Odds ratio of contact to services^a^																					
Logit	Contact out-patient psychiatrist					Contact psychologist				Contact GP-MHS				Psychiatric emergency clinic				Admission MH > 1 day			
	OR	*P*	CI		*N*	OR	*P*	CI		OR	*P*	CI		OR	*P*	CI		OR	*P*	CI	
*Family equivalent income* ^b^																					
Highest third	1				50,373	1				1				1				1			
Middle third	1.03	0.414	0.97	1.09		**0.71**	< **0.001**	**0.67**	**0.75**	**0.90**	< **0.001**	**0.86**	**0.95**	0.94	0.344	0.82	1.07	0.92	0.224	0.81	1.05
Lower third	**1.25**	< **0.001**	**1.17**	**1.34**		**0.49**	< **0.001**	**0.46**	**0.53**	**0.81**	< **0.001**	**0.77**	**0.86**	0.82	0.007	0.71	0.95	0.86	0.041	0.74	0.99
*Education (years)* ^b^																					
12+	1				48,183	1				1				1				1			
10–12	**0.92**	**0.005**	**0.86**	**0.97**		**0.67**	< **0.001**	**0.63**	**0.71**	**0.89**	< **0.001**	**0.85**	**0.94**	0.99	0.888	0.86	1.13	1.06	0.377	0.93	1.22
< 10	0.95	0.103	0.89	1.01		**0.37**	< **0.001**	**0.35**	**0.40**	**0.71**	< **0.001**	**0.67**	**0.75**	0.90	0.147	0.78	1.04	0.94	0.403	0.81	1.09

Incidence rate ratios of contact among patients who had one visit or more^a^																									
Poisson	Out-patient psychiatrist					Psychologist					GP-MHS					Psych. emergency clinic					Admission MH > 1 day				
	IRR	*P*	CI		*N*	IRR	*P*	CI		*N*	IRR	*P*	CI		*N*	IRR	*P*	CI		*N*	IRR	*P*	CI		*N*
*Family equivalent income* ^b^																									
Highest third	1				11,113	1				9033	1				17,638	1				1752	1				1783
Middle third	**0.90**	< **0.001**	**0.88**	**0.91**		**0.93**	< **0.001**	**0.91**	**0.95**		1.00	0.639	0.98	1.02		1.02	0.734	0.91	1.15		1.03	0.614	0.92	1.14	
Lower third	**0.83**	< **0.001**	**0.81**	**0.84**		**0.94**	< **0.001**	**0.91**	**0.96**		**0.94**	< **0.001**	**0.92**	**0.97**		1.06	0.364	0.94	1.14		0.95	0.382	0.84	1.07	
*Education (years)* ^b^																									
12+	1				10,659	1				8869	1				17,038	1				1677	1				1709
10–12	**0.92**	< **0.001**	**0.90**	**0.93**		**0.92**	< **0.001**	**0.90**	**0.93**		0.99	0.395	0.97	1.01		0.98	0.729	0.87	1.10		1.00	0.988	0.89	1.12	
< 10	**0.75**	< **0.001**	**0.74**	**0.76**		**0.80**	< **0.001**	**0.79**	**0.82**		**0.93**	< **0.001**	**0.91**	**0.96**		1.06	0.370	0.94	1.19		1.04	0.511	0.92	1.17	

Psychiatric emergency clinic includes emergency contacts and admissions up to 1 day

Results significant within a 95% confidence interval are marked in bold

*GP-MHS* mental health services by general practitioner (talk therapy), *Admission MH* admission to mental hospital, *IRR* incidence rate ratio, *CI* confidence interval, *P* 0.05

^a^Two separate analyses of correlation of income or education with mental health service used

^b^Adjusted for age, gender, country of origin, cohabitating status, access to vehicle, comorbidity psychiatric, comorbidity somatic

