# Supplementary material for: Impact of socioeconomic position and distance on mental health care utilization: a nationwide Danish follow-up study
Source: Soc Psychiatry Psychiatr Epidemiol. 2017 Aug 28;52(11):1405–13. doi: 10.1007/s00127-017-1437-2 (PMC5663810; doi:10.1007/s00127-017-1437-2)
Supplement: Supplementary file 1 — Supplementary material 1 (DOCX 24 kb) [file 127_2017_1437_MOESM1_ESM.docx]

***Supplement to method***

***Independent variables***

In this study, we used equivalent disposable family income, which adjusts the family income according to number of family members and children under the age of 25 years living at home after payment of taxes and interest. (The first adult counts as 1, each consecutive person aged >14 years as 0.5, and each person aged 0-14 years as 0.3. For a family of two adults and two children aged < 14 years, the household income will be divided by 2.1). The income was categorized into five groups according to quintiles.

**Extrinsic variables:**

*Comorbidity*

Diagnoses in the registers have been coded according to ICD-10 since 1994. The chronical diseases included: cancer, diabetes, psychiatric disorder, IHD, stroke, COPD and arthrosis. (ICD-10: C00 – C 43; C45 – C96; E10-E11; F 00 – F98.9; I20 – I25; I61 – I64; I69; J43 – J44; M05 – M06; M08-M09; M15). One occurrence in the register of one of the diagnoses counted as positive for a chronical condition. These diseases are categorized as diseases of public health concern in Denmark, which also includes osteoporosis and dementia. Osteoporosis was not included as symptoms are rarely known until the age of 70.(1) Dementia was not included for the same reason. Dementia is very rare for the present age group.

We excluded cancer as comorbidity if it had occurred ≥ 10 years before the first prescription and not since, since we then considered it to be cured. Likewise, a former psychiatric disorder was excluded as comorbidity if a person had only been registered with misuse of alcohol or drugs ≥ 10 years ago and had not since then been registered with a mental disorder. They too were considered to be cured.

The data for psychiatric comorbidity date back to 1969. Until 1994 the diagnoses were coded in ICD-8. The codes included were: 290; 292 – 301; 305 – 315. (Alcohol and drug misuse and sexual deviation were thus not included).

***Dependent variables***

*Information related to treatment*

GP-MHS is talk therapy provided by a GP. There is no formal requirement to the methods, except that it should be relevant .The GP has to receive regular supervision – from other GPs, psychologists or psychiatrists individually or in groups(2). Data were drawn from The Danish National Health Service Register for Primary Care, according to the codes in table S1 (3).

*Table S1: Codes for services provided in primary care.*

| **Type of health care service** | **Code in The Danish National Register for Primary Care** |
| --- | --- |
| GP-MHS (talk therapies) | 804003 +(804021-804027)+ (804247 – 804249) + 806101 |
| Psychologist contacts | (630110 – 630211) + (630214 – 630340) |
| Psychiatrist consultations | (240110 – 240140) + (240210 – 240236) + 241401 |

The public part of the expense for a psychologist (or a psychiatrist) is covered by the public health service system, which is also the case for insured persons. Thus privately insured persons were also included in our data. In 2013, 1.8 mill people (50% of Danes aged between 20 – 70 years) were covered by an additional private health insurance, and 13 million DKK (1.74 million €) were payed to cover expenses for psychiatrists or psychologists(4).

Reference list

(1) Vestergaard P, Rejnmark L, Mosekilde L. Osteoporosis is markedly underdiagnosed: a nationwide study from Denmark. Osteoporos Int 2005 February;16(2):134-41.

(2) RLTN. Forhandlingsaftale 2010 RLTN og PLO. 1-83. 21-12-2010.

Ref Type: Generic

(3) Andersen JS, Olivarius NF, Krasnik A. The Danish National Health Service Register. Scand J Public Health 2011 July;39(7 Suppl):34-7.

(4) forsikringogpension. Flere-end-to-millioner-danskere-har-nu-en-sundhedsforsikring.aspx. [www.forsikringogpension.dk](http://www.forsikringogpension.dk) . 11-1-2016.

Ref Type: Online Source
